# Transcriptional Reprogramming of *Staphylococcus aureus* in Chronic Rhinosinusitis Reveals a Persistence-Associated Phenotype

**DOI:** 10.3390/ijms27031429

**Published:** 2026-01-31

**Authors:** Lorena Tuchscherr, Stefan Monecke, Mateusz Jundzill, Martin Hölzer, Christian Brandt, Sindy Wendler, Juliane Priese, Ralf Ehricht, Orlando Guntinas-Lichius

**Affiliations:** 1 Institute of Medical Microbiology, Jena University Hospital, 07740 Jena, Germany; sindy.wendler@med.uni-jena.de; 2 Leibniz Institute of Photonic Technology, Leibniz Centre for Photonics in Infection Research (LPI), 07745 Jena, Germany; stefan.monecke@leibniz-ipht.de (S.M.); ralf.ehricht@leibniz-ipht.de (R.E.); 3 Center for Applied Research, InfectoGnostics Research Campus Jena, 07743 Jena, Germany; christian.brandt@med.uni-jena.de; 4 Center for Translational Medicine (CETRAMED), Jena University Hospital, Friedrich Schiller University Jena, 07747 Jena, Germany; 5 Institute for Infectious Diseases and Infection Control, Jena University Hospital, 07740 Jena, Germany; mateusz.jundzill@med.uni-jena.de; 6 Methodology and Research Infrastructure, Genome Competence Center (MF1), Robert Koch Institute, 13353 Berlin, Germany; hoelzerm@rki.de; 7 Institute of Physical Chemistry, Friedrich Schiller University Jena, 07745 Jena, Germany; juliane.priese@med.uni-jena.de; 8 Department of Otorhinolaryngology, Jena University Hospital, 07747 Jena, Germany; orlando.guntinas@med.uni-jena.de

**Keywords:** chronic rhinosinusitis (CRS), *Staphylococcus aureus* (*S. aureus*), chronic infections

## Abstract

Chronic rhinosinusitis (CRS) is a persistent inflammatory condition frequently associated with *Staphylococcus aureus*. The bacterium’s ability to evade immune clearance and establish long-term infection complicates treatment. In our previous study, we demonstrated that *S. aureus* isolates obtained from patients with CRS (CRS-*S. aureus* isolates; CSS) exhibit reduced glycolytic activity and cytotoxicity, which is consistent with a persistence-associated phenotype. Here, we present transcriptomic evidence that supports this shift. Comparative RNA sequencing of CSS and control (*S. aureus* isolates from healthy carriers, MIN) isolates from healthy individuals revealed significantly lower expression of genes involved in canonical virulence pathways in CSS isolates, particularly during the early growth phase. These profiles suggest reduced acute virulence in favour of metabolic changes that aid survival in the chronically inflamed sinus. The distinct transcriptional state of CSS isolates might reflect the influence of the CRS host milieu in shaping bacterial behaviour. Host factors such as sustained inflammation or altered nutrient availability may select for persistence-associated phenotypes. Together, these findings advance our understanding of chronic *S. aureus* infection and may aid/guide the development of therapies aimed at disrupting persistence programmes or enhancing host resilience.

## 1. Introduction

Chronic rhinosinusitis (CRS), a persistent inflammatory condition of the sinonasal mucosa, frequently involves colonisation by *S. aureus* strains exhibiting attenuated virulence traits, which likely contribute to bacterial persistence and disease chronicity. CRS is characterised by chronic mucosal inflammation, epithelial barrier dysfunction and dysregulated innate and adaptive immune responses. These factors create conditions that favour bacterial persistence over efficient clearance. In this context, *S. aureus* has repeatedly been implicated in chronic disease through mechanisms including biofilm formation, immune modulation and metabolic adaptation [1,2]

*Staphylococcus (S.) aureus* is a versatile opportunistic pathogen responsible for a wide range of acute and chronic infections. Isolates recovered from chronic or recurrent infections often exhibit reduced expression of cytotoxic and immunostimulatory factors. This phenotype has been associated with enhanced persistence and immune evasion, rather than with a diminished pathogenic potential. The association between reduced acute virulence and increased persistence is a well-established paradigm in bacterial pathogenesis, observed across various chronic infections [3,4].

Our previous study demonstrated that *S. aureus* isolates from CRS patients (CSS) display reduced glycolytic activity and diminished cytotoxicity compared to isolates from healthy carriers (MIN), suggesting metabolic and virulence trade-offs that favour survival within a chronically inflamed host environment [1,5,6]. These findings support the concept that CRS-associated *S. aureus* is characterised by a persistence-associated phenotype shaped by host-driven selective pressures, rather than by increased intrinsic virulence. However, the transcriptional mechanisms driving this phenotypic shift remain underexplored.

To elucidate these underlying regulatory changes, we performed RNA sequencing on two pairs of genetically related CSS and MIN isolates cultivated under standardised conditions. We focused on the early and intermediate growth phases (3 and 6 h) to capture dynamic gene expression patterns. The 3 h time point corresponds to the early exponential growth phase, during which virulence gene expression is rapidly induced. In contrast, the 6 h time point represents a transitional phase towards the post-exponential growth phase, enabling the stability and progression of transcriptional differences to be assessed. The novelty of this study lies in the integration of genome-wide transcriptional profiling with a conceptual framework of host fitness and disease tolerance, demonstrating how persistence-associated bacterial transcriptional states emerge in response to a chronically inflamed and immunologically compromised host niche. Our analysis focused on genes associated with virulence, immune evasion and biofilm formation. We hypothesised that CSS isolates would exhibit attenuated expression of the typical acute virulence pathway of canonical acute virulence programmes, alongside upregulation of metabolic and persistence-associated genes, reflecting adaptation to the ecological niche characterised by chronic inflammation. These insights aim to improve our understanding of *S. aureus* pathoadaptation in CRS, which has implications for the therapeutic targeting of chronic infections.

## 2. Results

### 2.1. Differential Transcriptional Profiles of CSS and MIN Isolates

**Figure 1:**
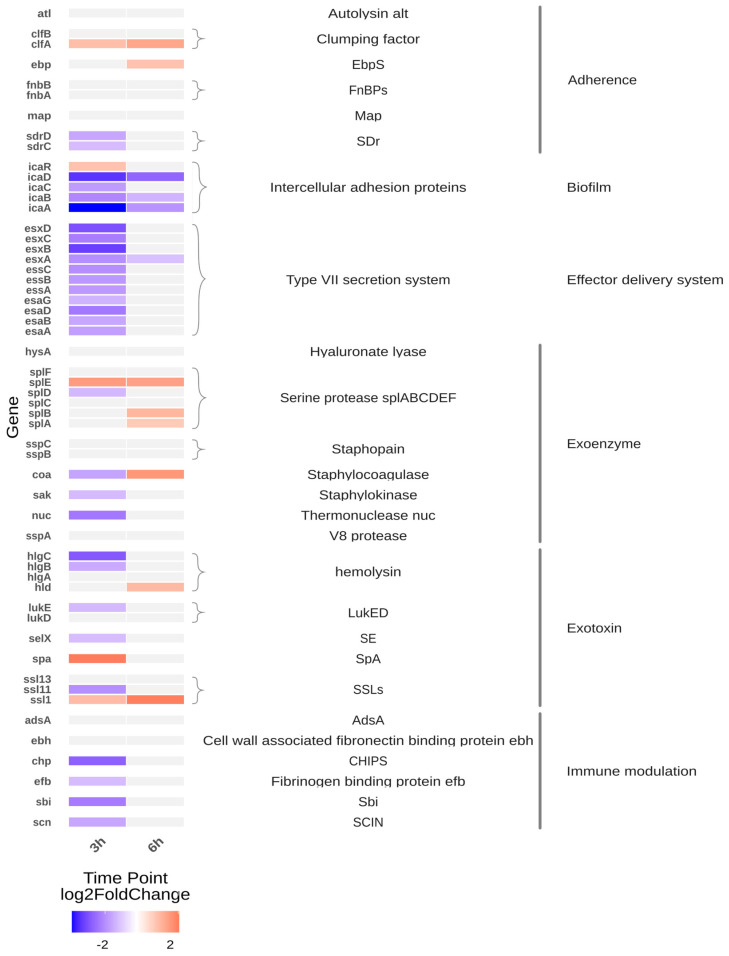
Heatmap of infection-related gene expression differences in CSS strains at 3- and 6 h post-incubation (CSS versus MIN). The heatmap displays the expression profiles of CSS strains in comparison to MIN strains at two time-points (3 h and 6 h). The selected genes and their functional groupings displayed in the figure were selected from *S. aureus* virulence factors annotated in the Virulence Factor Database (VFDB; [7]). Genes are grouped by functional categories, with colour intensity representing log_2_ fold changes in expression. A positive log_2_ fold change value indicates upregulation (increased gene expression), while a negative value indicates downregulation (decreased gene expression). Genes with expression changes below the threshold (|log_2_FC| < 1) are shown in light grey to de-emphasise non-significant changes that could be attributed to random variation.

The heatmap (Figure 1) depicts transcriptomic changes in *S. aureus* isolates after 3 and 6 h of cultivation in Tryptic Soy Broth (TSB). At 3 h, CSS isolates exhibited broad transcriptional repression across multiple virulence-associated pathways compared to isolates from healthy individuals (MIN). Notably, downregulated gene categories included components of the type VII secretion system, haemolysin-encoding genes (e.g., *hlgABC*), biofilm regulatory elements, exotoxin biosynthesis pathways, immune evasion factors (e.g., *scn*, *chp*), and *sdr* domain-containing adhesins.

As illustrated in Figure 1, several virulence-associated pathways exhibit significant transcriptional repression in CSS isolates after 3 h. However, regulation within individual virulence-associated gene groups was heterogeneous, with both increased and decreased expression observed for selected adhesins and toxin-related genes. After 6 h, most genes fall below the defined significance threshold (|log_2_FC| < 1), resulting in a light grey appearance. This suggests that the most significant differences between CSS and MIN isolates are evident during the initial growth phase, with transcriptional differences becoming less pronounced at later time points. These patterns indicate that not all virulence-associated genes are uniformly regulated, but rather that transcriptional changes are gene- and pathway-specific. Biofilm-associated regulatory elements remained comparatively upregulated after 6 h.

### 2.2. Upregulated Biological Processes Associated with CSS Isolates

**Figure 2:**
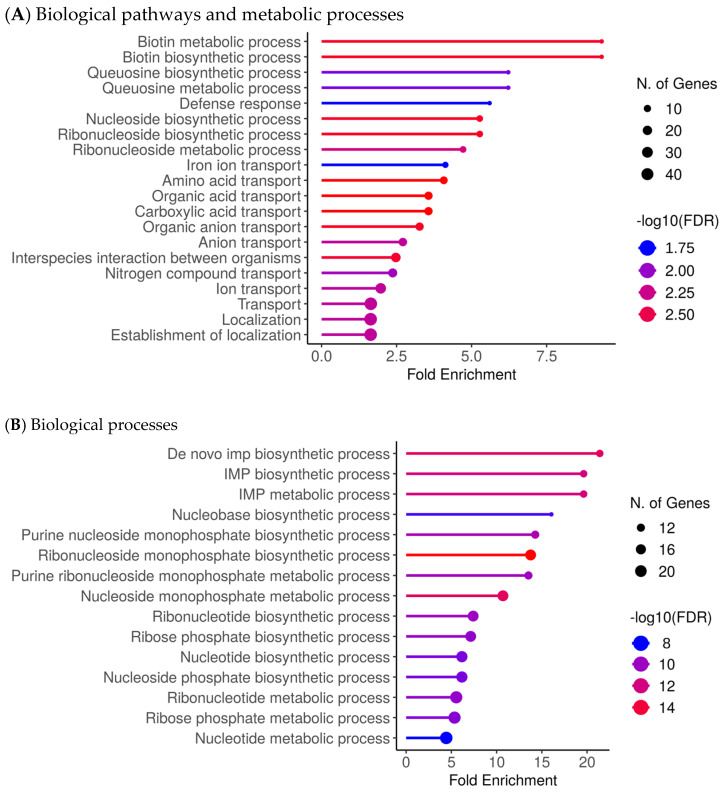
Upregulated biological processes and functional categories of *S. aureus* in CRS. (**A**). Biological pathways and metabolic processes were significantly enriched among transcripts upregulated in CSS isolates, including biofilm-associated functions, stress-response pathways, and persistence-related metabolic adaptations. (**B**). Biological processes are significantly enriched among transcripts downregulated in CSS isolates, primarily involving core biosynthetic and metabolic pathways. Enrichment significance thresholds: |log_2_FC| ≥ 1, adjusted *p* ≤ 0.05 (see Methods).

Figure 2 summarises enriched biological processes and functional categories associated with differential expression in CSS isolates (Figure 2; Supplementary Figures S1 and S2). Processes enriched among transcripts upregulated in CSS isolates were dominated by metabolic and transport-related functions, including biotin and queuosine pathways, nucleoside and ribonucleoside processes, iron ion transport, and multiple organic and amino acid transport categories (Figure 2A). Additional enrichment of stress-response, adhesion, and persistence- or virulence-associated functions was observed in the supplementary analyses (Figures S1A and S2A). By contrast, biological processes enriched among downregulated transcripts primarily involved core biosynthetic and metabolic pathways, particularly de novo IMP, nucleotide, and ribonucleotide biosynthesis (Figure 2B; Figures S1B and S2B).

Transcriptomic analyses of the CSS isolates further revealed a dual metabolic reprogramming characterised by strong upregulation of nutrient acquisition, cofactor biosynthesis, and salvage pathways, coupled with downregulation of biosynthetic growth functions. In particular, enriched upregulated pathways include biotin metabolism, queuosine biosynthesis/tRNA modification, nucleoside/ribonucleoside salvage, iron acquisition systems, and amino acid/carboxylic acid metabolic processes. Representative genes contributing to these enrichments included canonical genes such as *bioA*, *bioD*, *bioF*, *birA* (biotin pathway), *queD*, *queE*, *queC*, *tgt* (queuosine pathway), *deoD*, *ndk*, *adsA* (nucleoside salvage), *isdA*, *isdB*, *isdC*, *sbn*, *sstABCD* (iron uptake) and *ilvBCE*, *gltAB*, *sdhA*, *mdh* (amino acid/TCA/carboxylic acid metabolism) map onto the upregulated side. These genes are not displayed individually in Figure 2 but contribute to the enriched biological processes identified by functional enrichment analyses (Figure 2; Supplementary Figures S1 and S2). In contrast, pathways associated with de novo nucleotide and amino acid biosynthesis, central anabolic carbon assimilation, and certain energetic growth-associated processes were suppressed. Taken together, these data suggest that CSS isolates have a transcriptional profile characterised by reduced growth-associated biosynthetic activity and increased expression of pathways involved in resource acquisition.

## 3. Discussion

### 3.1. Attenuated Acute Virulence and Persistence-Associated Transcriptional States in CRS

The observation that CSS isolates display a lower expression of acute virulence determinants compared with isolates from MIN may at first appear paradoxical, since strains linked to clinical disease are often presumed to be more virulent. Importantly, this attenuation does not extend uniformly to all virulence-associated genes. Instead, Figure 1 reveals a nuanced transcriptional landscape in which only a subset of classical acute virulence determinants shows consistent downregulation. However, CRS is often linked to the formation of biofilms and/or the intracellular presence of *S. aureus*. Both factors have been associated with recalcitrance and persistence, even when toxin expression is reduced. Accordingly, when referring to “key virulence factors” in this study, we specifically denote canonical acute virulence programmes—such as cytotoxic toxins and secretion systems—that are central to host tissue damage and immune activation, rather than all genes traditionally annotated as virulence-associated.

This transcriptional pattern is consistent with the concept of pathoadaptation, whereby *S. aureus* modulates its transcriptional programme to enhance the persistence within the host [4,8]. Several studies have demonstrated that isolates derived from chronic or recurrent infections (as opposed to acute infections) frequently downregulate cytotoxic and immunostimulatory factors, such as haemolysins and toxins, while upregulating persistence-associated traits, including adhesion, biofilm formation, and immune evasion [9,10,11]. Previously, we demonstrated that isolates from CRS exhibit reduced glycolytic activity and diminished cytotoxicity [1]. These adaptations are considered metabolic and virulence trade-offs that facilitate long-term survival in the sinus cavity [1]. Similarly, clinical isolates with lower cytotoxic potential have been reported to survive in higher numbers and evade host clearance more efficiently than highly cytotoxic strains [11,12]. Persistence-associated phenotypes such as small-colony variants and *agr*-deficient isolates further exemplify this shift, as they are characterised by attenuated toxin production together with enhanced adhesion and biofilm development [8,13,14,15]. In the context of CRS, the downregulation of acute virulence determinants by CSS isolates may therefore represent a trade-off consistent with selection under chronic infection. By attenuating its aggressive host-damaging functions, *S. aureus* may evade immune clearance and establish itself in the chronically inflamed immunologically “unfit” mucosa of the sinuses [10,16].

### 3.2. Metabolic Reprogramming and Host-Imposed Selective Pressures

The metabolic reprogramming observed in CSS isolates is characterised by coordinated upregulation of nutrient acquisition, cofactor biosynthesis, and salvage pathways alongside suppression of energetically costly biosynthetic growth functions. Enhanced expression of biotin metabolism (including biotin biosynthesis and BirA-mediated regulation) has been linked to altered fatty-acid synthesis and membrane remodelling that support survival under limited exogenous cofactor availability, and BirA functions as both ligase and transcriptional regulator in *S. aureus* [17]. Enhanced expression of queuosine (Q) biosynthetic and tRNA-modifying genes is known to stabilise translation under stress and has been implicated in bacterial stress responses and biofilm phenotypes, consistent with adaptation to the inflammatory sinonasal niche [18,19]. Induction of nucleoside salvage pathways and extracellular nuclease/adenosine synthesis (e.g., *nuc*, *adsA*) permits nucleotide recycling and produces immunomodulatory adenosine, thereby both conserving metabolic resources and blunting host phagocytic responses—mechanisms that promote persistence [20]. Furthermore, strong upregulation of iron-acquisition systems (Isd family, staphyloferrin biosynthesis/transporters) is a canonical in vivo response to host nutritional immunity and directly enables bacterial survival in iron-restricted tissues [21]. Additional recent studies of *S. aureus* under iron-limited conditions (e.g., in the “xenometal–siderophore” conjugate study) also corroborate that *S. aureus* strongly induces siderophore biosynthesis and iron uptake when encountering nutritional immunity and downregulates iron-consuming systems (such as certain heme efflux or storage) to conserve resources [22]. Conversely, downregulation of energetically costly de novo biosynthetic pathways (purine/pyrimidine and some amino-acid synthesis routes) is a well-documented strategy that reduces metabolic burden, slows growth and increases tolerance to antibiotics, features typical of persister and chronic-infection states [23,24].

Together, these transcriptional patterns are consistent with survival under conditions of chronic inflammation and host-imposed nutrient limitation, which characterise the CRS sinonasal environment.

### 3.3. Host Fitness, Disease Tolerance, and Clinical Outcome

In the context of CRS, reduced expression of acute virulence determinants in CSS isolates likely reflects a trade-off consistent with selection under chronic inflammatory conditions. Attenuation of aggressive, host-damaging functions may limit immune activation, thereby facilitating persistence within the chronically inflamed, immunologically compromised sinus mucosa. These transcriptional alterations are consistent with enhanced bacterial persistence within the chronically inflamed sinonasal environment, potentially through reduced immune activation and improved nutrient utilisation efficiency. By contrast, MIN isolates expressed higher levels of acute virulence-associated determinants. We propose that this apparent paradox can be interpreted within a hypothesis-driven framework of host fitness, whereby, in a metabolically and immunologically “fit” host, *S. aureus* may engage virulence mechanisms primarily to ensure survival without necessarily triggering significant pathology. Conversely, in an “unfit” host, or in a host conditioned by chronic inflammation, even modest bacterial activity may contribute to disproportionate tissue damage.

Consistent with this interpretation, isolates from healthy carriers inhabit a metabolically and immunologically “fit”, hostile environment in the anterior nares, where stronger epithelial barriers and immune surveillance may necessitate higher expression of virulence genes to maintain survival and colonisation. Thus, the attenuated expression of acute virulence determinants observed in CSS isolates should not be interpreted as diminished pathogenic capacity, but rather as a hallmark of persistence-associated adaptation in the CRS niche following an acute phase in which this ecological niche was shaped. Accordingly, host factors would largely determine whether colonisation remains silent or progresses to a clinical infection. Persistence-associated adaptations would then accompany the transition from acute infection to chronic recurrent sinusitis (CRS) [25].

Because host immune and metabolic parameters were not directly assessed in this study, the proposed role of host fitness should be interpreted as a conceptual framework consistent with the observed bacterial transcriptional states rather than as a demonstrated causal mechanism.

### 3.4. Limitations of This Study

One limitation of the present study is that only two genetically related CSS-MIN isolate pairs were analysed. While this paired design enables controlled and biologically meaningful comparisons by minimising genetic background variability, it does not allow population-level inference. Consequently, our conclusions focus on the conserved transcriptional patterns observed within the studied lineages, rather than on their prevalence among all CSS S. aureus strains. The selected isolates belong to CC7 and CC15, which are well represented among human-associated S. aureus and are frequently reported in nasal carriage and clinical infection collections. This supports the clinical relevance of the findings.

Another limitation is that the transcriptomic observations rely solely on RNA-seq-based differential expression analysis and have not been independently validated using complementary approaches, such as qRT-PCR or functional assays. This issue should be addressed in future studies.

### 3.5. Outlook

These findings provide several perspectives for therapeutic innovation. For example, targeting the metabolic and translational mechanisms that enable *S. aureus* to adapt to the host, such as those involved in nutrient acquisition via siderophores, biotin biosynthesis or queuosine-associated translation pathways, may yield new antimicrobial strategies.

Furthermore, our results highlight the importance of considering host susceptibility as a critical factor in disease persistence. Understanding the host factors that facilitate bacterial adaptation could reveal ways to enhance patient resilience. Therefore, interventions aimed at restoring or enhancing host physiological ‘fitness’ could serve as a complementary approach, addressing both sides of the host–pathogen interaction rather than focusing solely on microbial eradication. Identifying risk factors that render individuals susceptible to CRS might also help prevent disease, for example, by eradicating *S. aureus* colonisation, in selected high-risk individuals may help prevent progression to clinical disease.

## 4. Materials and Methods

*S. aureus* isolates from chronic rhinosinusitis patients (CSS) and healthy individuals (MIN) were cultured overnight. The strains belonged to clonal complexes CC7 (CSS66 and MIN142) and CC15 (CSS126 and MIN93) that are both prevalent in Germany, and these isolates have been previously characterised [1]. Cultures were diluted in TSB broth to an optical density (OD_600_) of 0.05 the following day, and samples were collected at 3- and 6 h post-dilution. Total RNA was extracted using Quick-RNA Fungal/Bacterial Miniprep Kit (Zymo Research Corporation, Freiburg, Germany) at these time-points [26]. The quality of RNA was measured using Agilent 2200 TapeStation (Agilent Technologies, Santa Clara, CA, USA) with a cutoff value of RIN > 8, and quantity checked using Qubit 3 Fluorometer (Thermo Fisher Scientific, Darmstadt, Germany) and subjected to rRNA depletion using MICROBExpress Bacterial mRNA Enrichment Kit (Thermo Fisher Scientific, Darmstadt, Germany). Sequencing libraries were prepared using random hexamer primers that were used for cDNA synthesis and underwent RNA sequencing (RNA-seq) on an Illumina NovaSeq PE 150 device (San Diego, CA, USA). Differential gene expression analysis employed thresholds of |log_2_ fold change| ≥ 1 and an adjusted *p*-value ≤ 0.05 from DESeq2 analysis. The |log_2_FC| ≥ 1 threshold was selected to focus on transcriptional changes in sufficient magnitude to be biologically meaningful, a criterion commonly applied in bacterial RNA-seq studies to distinguish expression shifts from minor fluctuations. Gene annotation was performed using UniProtKB and UniParc accession mappings (UniProt Consortium, Hinxton, United Kindgdom).

The raw sequencing data were analysed using the RNAflow Nextflow workflow (version 1.4.7) [27,28,29]. In this workflow, quality trimming was performed using fastp, mapping was carried out using HISAT2 to the NCTC 8325 *S. aureus* genome (GCA_000013425.1), strand-specific counting was conducted on the gene level using featureCounts, and differential expression analysis between CSS vs. MIN and 3h vs. 6h groups was executed using DESeq2 [30]. Workflow was executed using default parameters. 

Functional enrichment of Gene Ontology (GO) biological processes and protein–protein interaction network clustering were conducted using ShinyGO v0.82Ge SX [31]. Virulence-associated genes were identified by cross-referencing the Virulence Factor Database (VFDB) [7]. Data visualisation comprised tree plots, lollipop charts, and network diagrams to highlight transcriptional patterns.

## 5. Conclusions

In conclusion, this study provides genome-wide transcriptomic evidence consistent with a persistence-associated transcriptional state in *S. aureus* isolates from patients with chronic rhinosinusitis. This state is characterised by attenuated expression of canonical acute virulence programmes, together with upregulation of metabolic pathways associated with nutrient acquisition and survival. The novelty of this work lies in the genome-wide characterisation of these persistence-associated bacterial transcriptional states and in their integration into a host-centred conceptual framework of fitness and disease tolerance, which is used here to contextualise—rather than directly demonstrate—the influence of host conditions on chronic infection.

Accordingly, the reduced expression of acute virulence determinants observed in CSS isolates should not be interpreted as diminished pathogenic potential, but rather as a persistence-associated transcriptional signature within a compromised, chronically inflamed sinonasal niche. These findings highlight the principle that virulence is not a fixed microbial characteristic, but rather a context-dependent outcome shaped by dynamic host–pathogen interactions. Classic persistence phenotypes, such as small-colony variants and *agr*-deficient strains, further exemplify host-driven selection of bacterial phenotypic states during chronic infection.

Together, these results refine our understanding of *S. aureus* pathoadaptation in CRS by emphasising the interplay between bacterial transcriptional plasticity and host susceptibility in determining disease outcomes. Although broader isolate sampling and independent functional validation will be required, the insights presented here may inform future therapeutic strategies aimed at targeting persistence-associated pathways and restoring host–microbe homeostasis to mitigate disease progression.

## Data Availability

The original contributions presented in this study are included in the article/Supplementary Material. The RNA-seq data generated in this study have been deposited in the Array Express database at EMBL-EBI under accession number E-MTAB-16578. Further inquiries can be directed to the corresponding author.

## Supplementary Materials

The following supporting information can be downloaded at: https://www.mdpi.com/article/10.3390/ijms27031429/s1.
