# Supplementary material for: Transcriptional Reprogramming of Staphylococcus aureus in Chronic Rhinosinusitis Reveals a Persistence-Associated Phenotype
Source: Int J Mol Sci. 2026 Jan 31;27(3):1429. doi: 10.3390/ijms27031429 (PMC12897772; doi:10.3390/ijms27031429)
Supplement: Supplementary file 1 [file ijms-27-01429-s001.zip › ijms-4066878-supplementary.pdf]

Supplemented material

Transcriptional Reprogramming of *Staphylococcus aureus* In Chronic Rhinosinusitis Reveals a Persistence-Associated Phenotype

Lorena Tuchscherr [1,*], Stefan Monecke [2,3,4], Mateusz Jundzill [5], Martin Hölzer [6], Christian Brandt [3,5], Sindy Wendler [1], Juliane Priese [7], Ralf Ehricht [2,3,4,7,†] and Orlando Guntinas-Lichius [8,†]

[1] Institute of Medical Microbiology, Jena University Hospital, 07740 Jena, Germany; lorena.tuchscherrdh@fluidect.com (L.T.) sindy.wendler@med.uni-jena.de

[2] Leibniz Institute of Photonic Technology, Leibniz Centre for Photonics in Infection Research (LPI), 07745 Jena, Germany; stefan.monecke@leibniz-ipht.de (S.M.); ralf.ehricht@leibniz-ipht.de (R.E.)

[3] Center for Applied Research, InfectoGnostics Research Campus Jena, 07743 Jena, Germany; christian.brandt@med.uni-jena.de (C.B.); stefan.monecke@leibniz-ipht.de (S.M.); ralf.ehricht@leibniz-ipht.de (R.E.)

[4] Center for Translational Medicine (CETRAMED), Jena University Hospital, Friedrich Schiller University Jena, 07747 Jena, Germany; stefan.monecke@leibniz-ipht.de (S.M.); ralf.ehricht@leibniz-ipht.de (R.E.)

[5] Institute for Infectious Diseases and Infection Control, Jena University Hospital, 07740 Jena, Germany; mateusz.jundzill@med.uni-jena.de

[6] Methodology and Research Infrastructure, Genome Competence Center (MF1), Robert Koch Institute, 13353 Berlin, Germany; HoelzerM@rki.de M.H.)

[7] Institute of Physical Chemistry, Friedrich Schiller University Jena, 07745 Jena, Germany; ralf.ehricht@leibniz-ipht.de (R.E.)

[8] Department of Otorhinolaryngology, Jena University Hospital, 07747 Jena, Germany; juliane.priese@med.uni-jena.de (J.P.); orlando.guntinas@med.uni-jena.de (O.G.-L.)

* Correspondence: lorena.tuchscherrdh@fluidect.com

† These authors contributed equally to this work.

GO biological process enrichment analysis of differentially expressed genes in *S. aureus* CSS vs. MIN (3 hours).

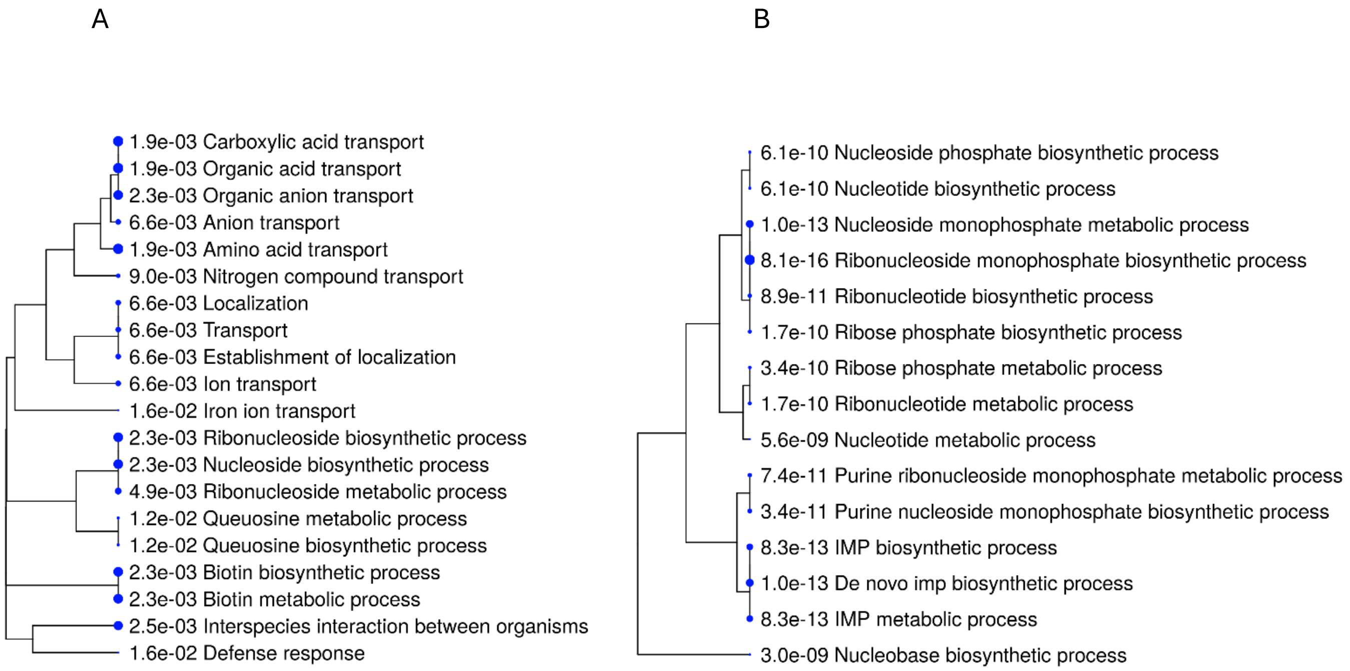

Figure S1: Hierarchical clustering tree of CSS *S. aureus* isolates. Gene Ontology (GO) biological process enrichment displayed as a hierarchical clustering tree of significantly upregulated genes (Fig. S1 A) and downregulated genes (Fig. S1B). P-values are shown to the left of each term. (A) Enriched processes among upregulated genes include carboxylic acid and organic acid transport, anion and amino acid transport, various localization and ion transport processes, and biosynthetic pathways such as ribonucleoside, nucleoside, queuosine, and biotin metabolic processes, along with terms related to interspecies interactions and defense response. P-values indicate the statistical significance of enrichment for each term. (B) Downregulated genes show strong enrichment for pathways associated with nucleoside and nucleotide biosynthesis, including nucleoside phosphate biosynthetic process, ribonucleoside monophosphate biosynthesis, ribose phosphate biosynthesis, as well as nucleotide and ribonucleotide metabolic processes. Additional enriched terms include purine ribonucleoside and nucleoside monophosphate metabolism, IMP biosynthesis, and de novo IMP biosynthetic process.

Increased carboxylic/organic acid, amino acid and anion transport, and ion transport is typical of bacteria adapting to fluctuating nutrients, pH and ionic stress in inflamed mucosa and biofilm environments, supporting survival under host immune pressure and intermittent antibiotic exposure.Upregulated biosynthetic processes for ribonucleosides, queuosine and biotin are linked to maintaining translation fidelity, stress tolerance and cofactor supply, which can enhance bacterial persistence and resilience rather than simply growth rate. Enrichment of "interspecies interaction" and "defense response" terms indicates active sensing of and response to host immunity and competing microbes, consistent with a pathobiont role for staphylococci that modulate and withstand host inflammation in CRS mucosa.

Reduced expression of nucleoside/nucleotide and IMP de novo biosynthetic pathways is compatible with a lower-replication, resource-conserving state often seen in chronic, biofilm-embedded staphylococci that rely more on salvage pathways than on energetically expensive de novo synthesis. Such downregulation is associated in other persistent *S. aureus* contexts with increased antibiotic tolerance and long-term

survival, rather than acute virulent expansion, fitting the clinical picture of long-standing CRS with recurrent but often subacute symptoms.

Our pattern—enhanced transport, stress and interaction processes with dampened de novo nucleotide biosynthesis—fits this model of a metabolically specialized, persistence-oriented staphylococcal population, suggesting that in CRS these bacteria act as chronic pathobionts that help sustain mucosal inflammation rather than behaving primarily as rapidly dividing, acute pathogens. The data support viewing staphylococci in CRS as adapted to a chronic, inflammatory niche, implying that eradication may require strategies targeting biofilms, metabolic vulnerabilities (e.g., cofactor or transport systems) and host–microbe interactions, rather than only traditional growth-inhibitory antibiotics (1-2).

A

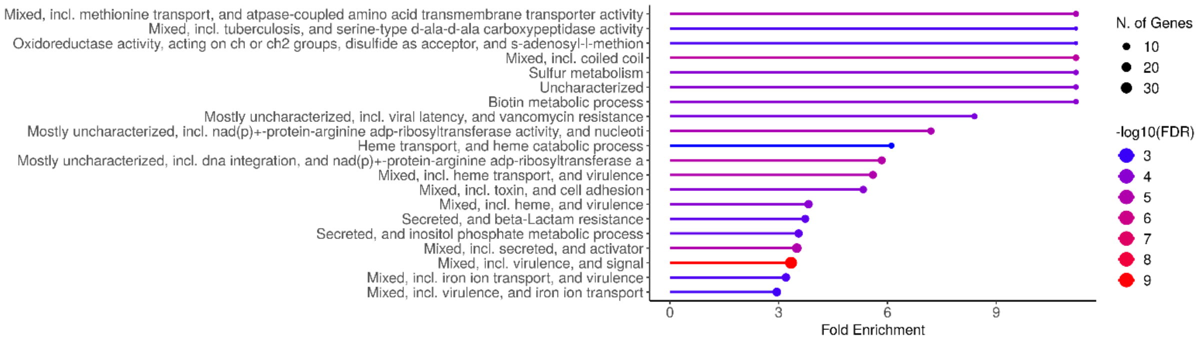

B

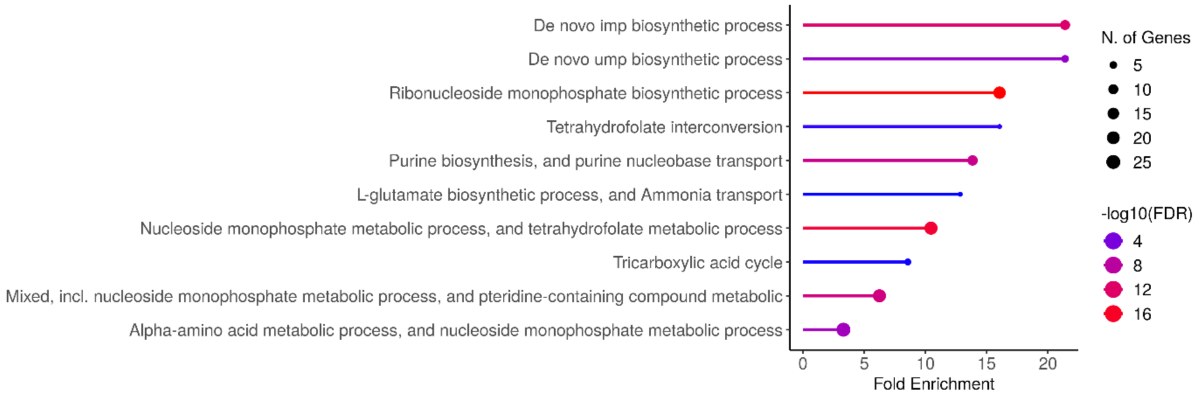

Figure S2: **Lollipop chart of CSS *S. aureus* isolates**. Gene Ontology (GO) biological process enrichment displayed as a lolipop chart. Pathway and functional category enrichment of upregulated genes (A) or downregulated genes (B) shown as a ranked dot plot. The x-axis represents fold enrichment, dot size

indicates the number of genes in each category, and dot color denotes statistical significance (–log10(FDR)). A. Upregulated genes are enriched in transport systems, sulfur and biotin metabolism, redox-associated activities, cell adhesion, toxin and heme metabolism, β-lactam resistance–associated functions, and mixed virulence-associated categories. Collectively, these enriched pathways reflect complex metabolic and virulence remodeling consistent with bacterial adaptation to the chronic host environment. B. Processes with the highest fold enrichment include de novo IMP biosynthetic process, de novo UMP biosynthetic process, ribonucleoside monophosphate biosynthetic process, and pathways related to folate metabolism, amino-acid metabolism, and nucleotide metabolism. Together, these results demonstrate that downregulated genes are predominantly involved in nucleotide biosynthesis, central carbon metabolism, and amino-acid metabolic pathways.

References:

1. Goldie, S. P., Lau, L. C., Jones, H. A. S., Harries, P. G., Walls, A. F., & Salib, R. J. (2025). Identification of Novel *Staphylococcus aureus* Core and Accessory Virulence Patterns in Chronic Rhinosinusitis. *International Journal of Molecular Sciences*, *26*(8), 3711. https://doi.org/10.3390/ijms26083711

2. Houtak G, Bouras G, Nepal R, Shaghayegh G, Cooksley C, Psaltis AJ, Wormald PJ, Vreugde S. The intra-host evolutionary landscape and pathoadaptation of persistent *Staphylococcus aureus* in chronic rhinosinusitis. Microb Genom. 2023 Nov;9(11):001128. doi: 10.1099/mgen.0.001128.
